# Lighting Up Neural Circuits by Viral Tracing

**DOI:** 10.1007/s12264-022-00860-7

**Published:** 2022-05-16

**Authors:** Liyao Qiu, Bin Zhang, Zhihua Gao

**Affiliations:** 1 Department of Neurobiology and Department of Neurology of the Second Affiliated Hospital, NHC and CAMS Key Laboratory of Medical Neurobiology, Zhejiang University School of Medicine, Hangzhou, 310058 China; 2 The MOE Frontier Science Center for Brain Research and Brain-Machine Integration, School of Brain Science and Brain Medicine, Zhejiang University, Hangzhou, 310058 China

**Keywords:** Viral tracing, Neural circuit, Anterograde, Retrograde, Transsynaptic

## Abstract

Neurons are highly interwoven to form intricate neural circuits that underlie the diverse functions of the brain. Dissecting the anatomical organization of neural circuits is key to deciphering how the brain processes information, produces thoughts, and instructs behaviors. Over the past decades, recombinant viral vectors have become the most commonly used tracing tools to define circuit architecture. In this review, we introduce the current categories of viral tools and their proper application in circuit tracing. We further discuss some advances in viral tracing strategy and prospective innovations of viral tools for future study.

## Introduction

The human brain, consisting of nearly 86 billion highly interwoven neurons, is the most complex and sophisticated organ that instructs versatile physiological functions and behaviors. Each neuron contacts or is contacted by tens of thousands of other neurons *via* synapses, by which neurons transmit information from one to another. These neurons connect with each other to constitute intricate functional networks, namely neural circuits, to precisely transmit and process information in the brain. Unraveling the complex organization of these circuits is crucial to decipher how information is processed and how instructions are executed to generate thoughts and guide versatile behaviors.

A number of techniques have been developed to define the architecture of neural circuits. Fifty years have passed since the first use of horseradish peroxidase as a retrograde tracer in 1971 [1, 2]. Over the past half century, many new chemical tracers have been developed, for example, the anterograde tracer *Phaseolus vulgaris*-leucoagglutinin [3] and dextran-amine [4], and the retrograde tracers Fluoro-Gold [5] and cholera toxin B subunit [6], which did help to characterize the overall architecture of neural circuits. However, labeling strategies using these chemical tracers are usually transient with no cell-type-specificity. In recent years, the development of viral tracers has rapidly moved forward the dissection of the neural circuit. Since the first use of Herpes simplex virus (HSV) in neuroanatomical tracing in 1974 [7], engineered viral vectors have become the most commonly-used tools in neural circuit mapping, due to reduced cytotoxicity, long-term expression of reporter genes, efficient axonal transport or transsynaptic transport, cell-type-specific access and spatiotemporal transduction in a genetically modified background. Combined with the Cre/Flp-mediated recombination strategy, viruses containing fluorescent protein expression cassettes can selectively trace neuronal somas, their projections, and their synaptically connected neurons, thus lighting up the neural circuits in the brain.

Viruses that are routinely used in neural tracing differ in tropism, axonal or transsynaptic transport, and transgene expression (Table 1). Therefore, it is important to understand the characteristics of different viruses in order to choose the right tools for different purposes. In this review, we introduce the key features of commonly used viral vectors and their appropriate application for different experimental needs, along with recent progress and prospects in the development of advanced viral tracers to meet more needs and resolve complicated problems.

**Table 1:** Key characteristics of viruses commonly used in viral tracing.

Virus	Genome type and size	Payload capacity (kb)	Spread direction	Transsynaptic	Integration into genome	Onset and duration of expression
HSV	dsDNA ~ 152 kb	30–40	Anterograde Retrograde	Yes	No	Onset: hours Duration: 5–7 days
AAV^#^	ssDNA ~ 4.7 kb	~ 4.7	Anterograde (except AAVrg)	No (except AAV1 and AAV9)	No	Onset: ~1 week Duration: months
CAV-2	dsDNA 32 kb	~ 30	Retrograde	No	No	Onset: days to weeks Duration: months
RV	(−) ssRNA ~ 12 kb	3.7–4	Retrograde	Yes	No	Onset: ~2 days Duration: months
PRV	dsDNA ~ 142 kb	30–40	Retrograde	Yes	No	Onset: hours Duration: variable

dsDNA, double-stranded DNA; ssDNA, single-stranded DNA; ssRNA, single-stranded RNA; (−), negative sense; HSV, Herpes simplex virus; AAV, adeno-associated virus; AAVrg, rAAV2-retro; CAV-2, canine adenovirus 2; RV, rabies virus; PRV, pseudorabies virus; kb, kilobase. ^#^AAV has many serotypes including AAV1, AAV2, AAV5, AAV8, and AAV9. All AAV types are anterogradely transported except AAVrg, which is retrogradely transported. Only AAV1 and AAV9 can spread transsynaptically, whereas the other AAVs cannot

## Viral Tools in Neural Tracing

A century ago, people suffered from a disease characterized by blisters in the oral and genital regions. The arch-criminal of this disease was unknown until the identification of HSVs in 1923 [8]. HSVs infect skin epithelial cells, spread through the sensory nerves, and finally reach the neuronal perikarya in the central nervous system [9]. These discoveries inspired scientists to test the possibility of applying HSVs to circuit tracing in the 1980s [10, 11]. Since then, more types of viruses have been identified and their genomes further genetically modified in the lab to generate recombinant viruses. These viruses have been widely used in neural tracing and promoted the dissection of neural circuits.

**Fig. 1:**
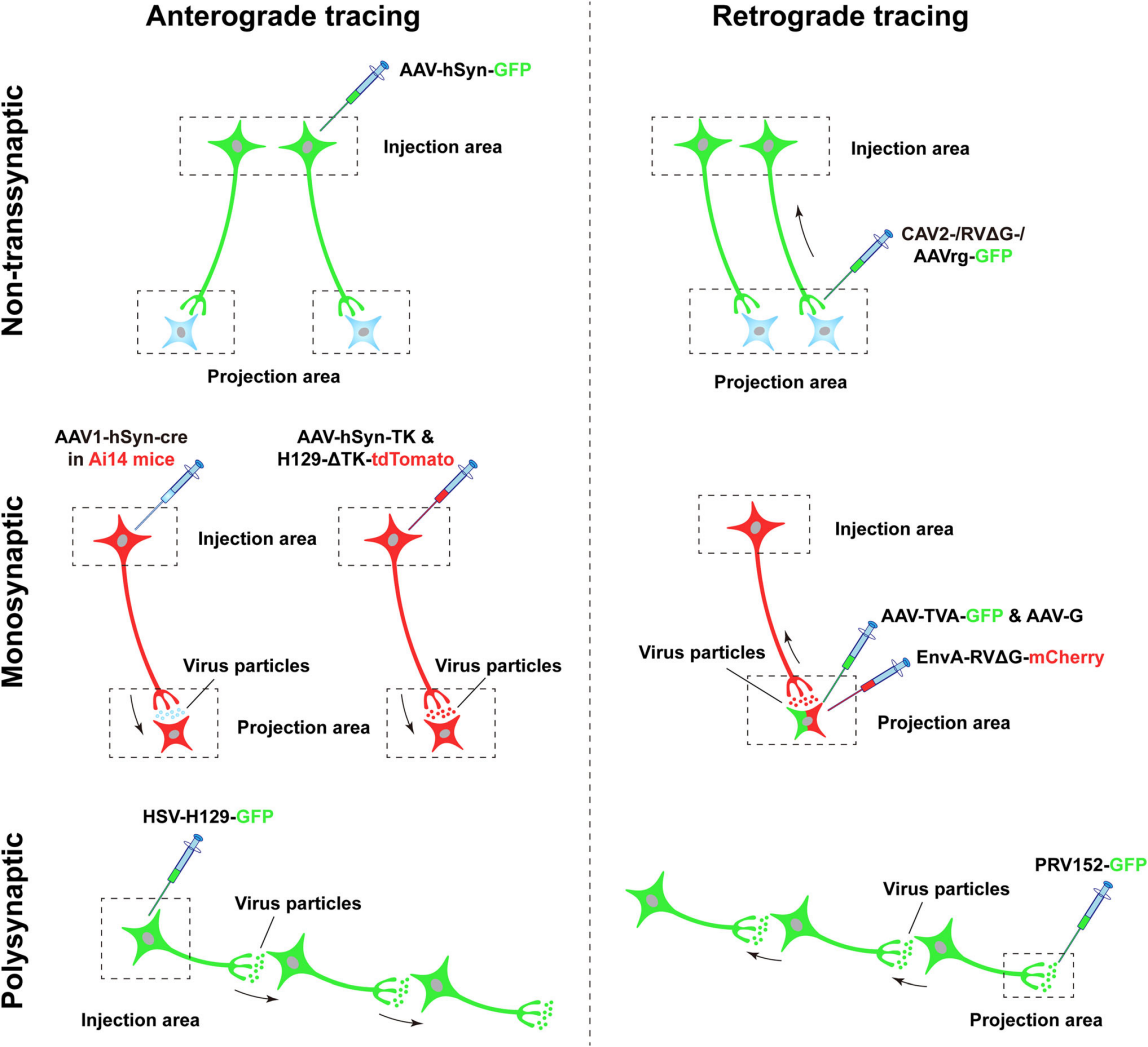
Schematic of different viral-tracing strategies. Viral-tracing strategies can be divided into three classes based on their ability to cross synapses: non-transsynaptic (upper), monosynaptic (middle), and polysynaptic (lower). Each class contains both anterograde (left panels) and retrograde (right panels) approaches based on the transport direction of viruses. In anterograde tracing, viruses are often injected into the somal region (injection area), infecting the somas and fully labeling the axonal terminals by expression of fluorescent proteins, thus tracing the terminal regions (projection area). The non-transsynaptic viruses are unable to cross synapses (upper left), whereas the monosynaptic or polysynaptic viruses can transfer to the downstream neurons spanning one (middle left) or multiple synapses (lower left). In non-transsynaptic retrograde tracing, viruses are usually injected into the terminal region, in which they infect the axon terminals and spread retrogradely to the somas (upper right). In monosynaptic or polysynaptic retrograde tracing, viruses are injected into the postsynaptic neuronal areas, are transferred to the presynaptic terminals, and spread retrogradely to the somas (middle right) or to further upstream synaptically-connected neurons (lower right). The neurons that are in both green and red indicate the co-expression of GFP and mCherry, while blue neurons are not infected by viruses (the same convention is used in the following figures). The black arrows indicate the spread direction of viral particles. AAV, adeno-associated virus; AAV1, one subtype of AAV; AAVrg, a retrograde-tracing variant of AAV; CAV, canine adenovirus; hSyn, human Synapsin I; TK, thymidine kinase; HSV, Herpes simplex virus; H129-ΔTK, a TK-deleted anterograde-tracing recombinant of HSV; G, rabies glycoprotein; RVΔG, G-deleted rabies virus; EnvA, avian ASLV type A envelope protein; TVA, avian receptor for EnvA; PRV152, a retrograde-tracing recombinant of the pseudorabies virus.

Several types of viral tracer are routinely used, including the HSV, adeno-associated virus (AAV) [12], canine adenovirus-2 (CAV-2) [13], rabies virus (RV) [14], pseudorabies virus (PRV) [15]. In general, based on their ability to cross the synapses, viral tracers can be categorized into two classes: non-transsynaptic and transsynaptic. The non-transsynaptic viruses are unable to span synapses to other neurons and are restricted to infected neurons, whereas the latter can cross synapses and spread to other synaptically-connected neurons. Both classes contain viruses that are transported in the anterograde or retrograde direction along axons. Detailed information regarding the different properties and applications of viruses has been extensively reviewed [16–20]. Here, we summarize the features of widely-used viruses in neural tracing (Fig. 1).

## Non-transsynaptic Tracing Virus

As noted above, non-transsynaptic viruses are unable to cross synapses and are restricted to locally infected neurons. Based on the directionality of viral transport along axons, non-transsynaptic viruses are further divided into anterograde and retrograde viruses. Anterograde viruses generally infect neuronal somas and viral transgene products such as fluorescent proteins are transported from neuronal somas to the axonal terminals, whereas retrograde viruses usually infect nerve terminals and are transported from the terminals to the somas.

### Anterograde Viral Tracers

Non-transsynaptic anterograde tracing requires viruses to infect neuronal somas and fully label their axonal terminals either by the anterograde transport of viral particles or their passive diffusion along axonal processes. With the expression of reporter genes from the virus and subsequent filling throughout the neuronal soma and processes with fluorescent proteins, anterograde tracing is able to determine the output of a certain neural pathway and delineate neuronal morphology. While many types of viruses meet this need, AAVs are the most extensively used anterograde tracing tools [21]. AAVs are non-enveloped single-stranded DNA viruses with a gene payload capacity limited to ~ 4.7 kb. Several advantages make AAVs the most popular tools in neural tracing. First, AAVs cannot self-replicate without a helper, therefore their expression is generally restricted to the injected neurons. Second, AAVs are rarely integrated into the host genome and their immunogenicity is low, with rare immune responses or toxicity [22]. Third, transgenes in AAVs are persistently and stably expressed for several months [23].

AAVs have multitudes of serotypes, defined as viral capsids with diverse antigenicity. Different AAV serotypes bind to different receptors expressed by different cell populations, resulting in species-, tissue- and cell-specific tropisms [24]. The most commonly used recombinant AAV serotypes include AAV1, AAV2, AAV5, AAV8, and AAV9 [25]. Since the diffusion of AAV2 is limited and it is highly selective for neurons [26], recombinant AAV (rAAV) vectors currently in use are based on the framework of AAV2. rAAVs are constructed by packaging the genes of interest flanked by two inverted terminal repeat sequences of AAV2 with the capsids of other serotypes [27] such as AAV8 or AAV9 to make a hybrid AAV2/8 or AAV2/9. These engineered rAAVs combine the advantages of different serotypes and meet different needs in studies that require different degrees of viral diffusion.

Different AAV serotypes have different cell tropisms. For example, AAV5 appears to exhibit a glial tropism in primary cultures of rat cortical cells [28], while others are more selective for neurons. However, the serotype is not the main determinant of cell type-specific infection *in vivo*, because the brain regions and how the virus is administered also affect the tropism. Therefore, specific promoters are required for AAVs to express transgenes in particular cell types. For example, hSyn (human Synapsin I) is commonly used as a pan-neuronal promoter and gfaABC_1_D as an astrocyte-specific promoter [29]. The most common way to selectively target certain cell types is *via* the Cre-LoxP (locus of x-over P1 site) and the Flp-FRT (Flp recombination target site) system-based genetic approaches, by which transgene expression is allowed in the presence of Cre or Flp recombinases [30, 31].

### Retrograde Viral Tracers

When studying the function of a certain brain region in neural circuits, it is indispensable to identify the source of upstream inputs, and this can be achieved by retrograde tracing. Retrograde tracing is based on viral entry from the axonal terminals and retrograde transport of the viral particles to the neuronal somas. In contrast to anterograde tracing, retrograde tracing requires viral binding with surface receptors expressed at the axonal terminals. Several types of virus exhibit the properties of terminal entry and retrograde spread, including CAV-2, RV, PRV, and some specific strains of HSV. Among these viruses, only CAV-2 cannot be transmitted across synapses while the last three can (see below). CAV-2 is a double-stranded DNA adenovirus with many advantages such as little immunogenicity, a relatively large gene payload of up to 30 kb, high selectivity for neurons, enduring gene expression, and efficient retrograde transport [13, 32, 33]. However, the drawback that limits its application is cell tropism. The binding and endocytosis of CAV-2 require the coxsackie adenovirus receptor (CAR), which is predominantly localized at presynaptic terminals and mediates viral entry and retrograde transport [34, 35]. In other words, CAV-2 exclusively infects axon terminals that express CAR. Therefore, retrograde tracing using CAV-2 may fail to label neurons with low or no CAR expression.

**Fig. 2:**
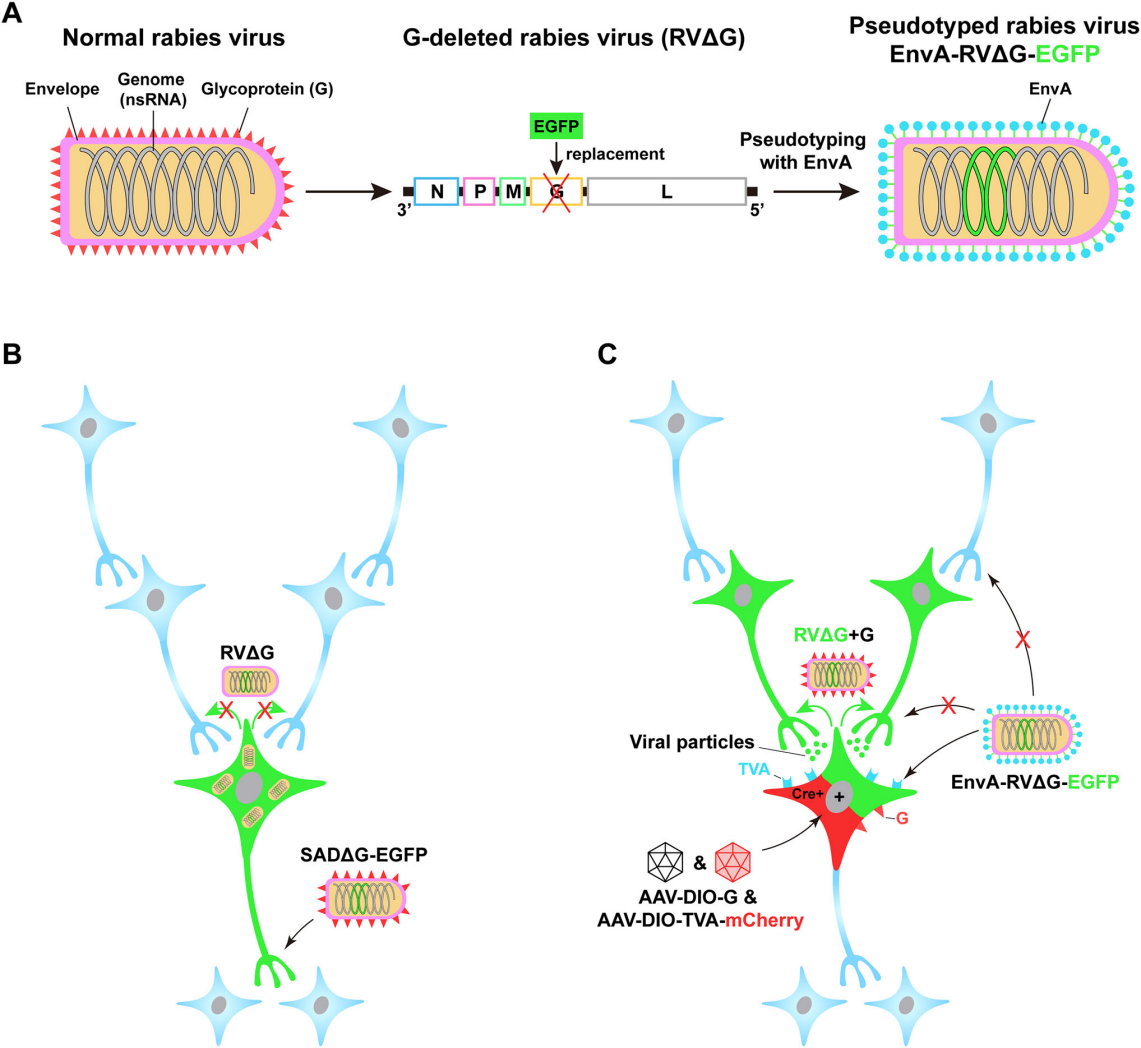
Pseudotyped rabies virus for retrograde tracing. **A** Engineering rabies virus (RV) by glycoprotein deletion and EnvA pseudotyping. Normal RV (left) contains a negative-strand RNA genome consisting of five genes and an envelope that is coated with the glycoprotein (G), which is coded by one of the five genes. RV can be engineered by replacing the G gene with an enhanced green fluorescent protein (EGFP) and pseudotyping this G-deleted RV with EnvA, the envelope protein of an avian virus (middle and right). **B** Non-transsynaptic retrograde tracing using SADΔG-EGFP. The recombinant RV SADΔG-EGFP, in which the G-coding gene is deleted, has been coated with G but loses the ability to produce G. This virus can infect axon terminals (shown by the black arrows without red crosses) and retrogradely spread to the somas. It retains the ability to replicate and produce a large amount of virus, thereby enhancing the EGFP fluorescent signal. Unable to synthesize G, however, the newly produced offspring fail to spread to synaptically-connected neurons (shown by the green arrows with red crosses). **C** Monosynaptic retrograde tracing using EnvA-pseudotyped RV. Due to the absence of endogenous receptors for EnvA, EnvA-RVΔG is unable to infect neurons in the mammalian brain (shown by the black arrows with red crosses). When the EnvA receptor, TVA, is exogenously expressed in the Cre^+^ neurons *via* Cre-dependent (DIO) AAV helper vectors, EnvA-RVΔG can selectively infect the TVA-harboring cells (neurons in green plus red), which is shown by the black arrows without red crosses. With the complementation of G in the same cells, the newly generated virus, RVΔG+G regains the ability to transsynaptically spread (shown by the green arrows) to presynaptic neurons (green). Due to the absence of G expression in these presynaptic neurons, however, the virus is unable to further spread out of these cells (blue). The red cross means inability. ns, negative strand; N, nucleoprotein, P, phosphoprotein; M, matrix protein; G, glycoprotein; L, the polymerase of rabies virus; DIO, double-floxed inverse open reading frame; SADΔG, a G-deleted RV strain.

Although RV naturally exhibits the property of transsynaptic transport and is most commonly used in retrograde monosynaptic tracing (see below), a newly-engineered RV with glycoprotein (G)-deleted (SADΔG-EGFP) is also an excellent tool for non-transsynaptic retrograde tracing (Fig. 2A). In this RV, the gene encoding the glycoprotein is substituted by the gene encoding the enhanced green fluorescent protein (EGFP) [36]. As the envelope glycoprotein is essential for the RV to cross synapses, the recombinant RV loses the ability to spread to other synaptically-connected neurons and is confined to the initially infected cells (Fig. 2B). However, since the recombinant virus retains the ability to replicate, the amplified virus contributes to enhanced fluorescent signals, making it suitable for delineating the morphology of retrogradely-labeled neurons.

Another powerful tool for non-transsynaptic retrograde tracing is rAAV2-retro, a recently developed AAV variant that exhibits a much higher efficacy of retrograde transport than other AAV serotypes and CAV-2 [37]. This variant contains a mutant capsid generated by error-prone PCR that demonstrates the highest potency of retrograde transport after several rounds of selection. The rAAV2-retro has highly-efficient retrograde transportability with stable transgene expression and has become the most commonly used retrograde viral tracer in dissecting projection-specific neural pathways. Recently, by injecting this powerful viral tracer into the posterior pituitary (PPi), we successfully labeled the neuroendocrine cell ensemble projecting to the PPi and reconstructed the three-dimensional architecture of the hypothalamo-neurohypophysial system [38]. Other than rAAV2-retro, additional engineered AAVs with high efficiency of retrograde transport, including AAV MNM008 [39], AAV2 R585/R588 [40], and AAV-TT [41], have been developed. Among these viruses, AAV MNM008 appears to exhibit improved retrograde infectivity of dopaminergic neurons [39]. The ability to carry fluorescent protein expression cassettes, along with the properties of efficient retrograde transport and low toxicity make rAAV2-retro an ideal tool for retrograde tracing in modern neuroscience research.

## Transsynaptic Tracing Virus

Transsynaptic tracing viruses cross synapses and spread to other neurons in neural circuits mostly owing to their self-replication. Based on their spreading direction between pre- and post-synaptic neurons, transsynaptic viruses are also divided into anterograde (from pre- to post-synaptic neurons) and retrograde (from post- to pre-synaptic neurons) viruses.

### Anterograde Transsynaptic Viral Tracers

Neurons are intricately connected with each other *via* synapses, constituting a complex hierarchical network. To fully understand the architecture of the neural circuits, it is necessary to dissect the anatomical organization of synaptic pathways. Although AAVs are wonderful tools for delineating neuronal morphology and projection areas, they do not reveal synaptic connections between neurons in different regions. Neither do they provide information regarding the molecular identity of the downstream innervated neurons. Therefore, anterograde transsynaptic viral tracers are required to resolve these problems.

Several types of the virus naturally exhibit transsynaptic transduction, due to their self-replication in the initially infected neurons and spread to the downstream neurons. One of them is HSV, an enveloped double-stranded DNA virus with a quite large capacity for transgene packages compared with other viruses, with a gene payload ~ 30–40 kb. HSV has many strains that exhibit different properties of anterograde and retrograde transsynaptic transport with distinct cytotoxicity [42, 43]. H129, one strain of HSV type 1, has been widely used in anterograde multisynaptic neural tracing [44], especially in studies that define the peripheral afferent or efferent pathways of some brain regions [45]. However, since H129 is a replication-competent virus, it is able to continuously spread across multiple levels of synapses. This leads to uncontrollable infection in the brain, which may exacerbate cytotoxicity and eventually lead to the death of the animal. Moreover, it is difficult to distinguish the neurons with monosynaptic and polysynaptic connections. Another limitation is that native HSV cannot selectively label specific neuronal populations, but this has recently been resolved by engineering the HSV genome. In the engineered virus, the thymidine kinase (*TK*) gene, required for viral replication, is replaced by a cassette including *loxP-STOP-loxP* with downstream *tdTomato* and a codon-modified *TK* gene [46]. This recombinant HSV, namely H129ΔTK-TT, is unable to express the *TK* gene and loses the ability of replication and transsynaptic spread in the absence of Cre recombinase. The virus restores the replication competence and drives the expression of the reporter gene only in the Cre-containing neurons and their postsynaptic neurons, thereby making it a good tool for anterograde tracing in combination with Cre-based genetic animal strains. Another recombinant H129 (H129-ΔTK-tdT) has been generated by replacing the viral *TK* gene with the cytomegalovirus (CMV) promoter-driven *tdTomato* gene, to allow reporter gene expression but disrupt the ability to replicate and spread across synapses [47]. When an AAV helper vector carrying the *TK* cassette is present in H129-ΔTK-tdT infected cells, H129-ΔTK-tdT restores the ability to replicate and transduce the postsynaptic neurons. However, H129-ΔTK-tdT cannot continue to spread into downstream neurons due to the lack of TK expression in the second-order neurons, making the anterograde tracing strictly monosynaptic. In combination with Cre-dependent AAV helpers, this H129-ΔTK virus has been successfully used to identify a population of neurons in the Edinger-Westphal nucleus that is directly innervated by Htr2c neurons in the ventral CA1 [48].

Although HSV1 is a powerful tool in anterograde transsynaptic tracing, its high toxicity limits its applicability in the functional dissection of specific neuronal connections for prolonged periods. In addition, HSV is also taken up by axon terminals and exhibits delayed retrograde transport [46]. Recently, a live attenuated vaccine for yellow fever (YFV), YFV-17D, has been engineered for anterograde transsynaptic tracing with low cytotoxicity [49]. This YFV-17D contains three structural protein-encoding genes (C, prM, and E) and five genes (NS1–NS5) that are crucial for viral replication. By deleting the NS1 gene, YFV^ΔNS1^ loses the ability to replicate. After complementing NS1 in the starter neurons and postsynaptic neurons, YFV^ΔNS1^ recovers the ability to spread transsynaptically and label the postsynaptic neurons. Similar to HSV, however, the YFV virus also exhibits delayed retrograde transport. To limit its retrograde transport, the authors used the inducible Tet-ON strategy to temporally restrict the complementation of NS1 by intermittent doxycycline induction to minimize viral replication, thereby reducing the retrograde transport and cytotoxicity. Moreover, by deleting the structural protein-encoding genes (C, prM, and E), the new variant, YFV^ΔCME^, is unable to package itself, thus losing the ability to infect neurons. YFV^ΔCME^ viruses recover the packaging ability in the starter cells when the structural proteins are provided and possess the ability to spread to postsynaptic neurons. However, due to the absence of the structural proteins in the downstream neurons, the viruses fail to transfer further, thereby achieving monosynaptic anterograde tracing. Also, since the YFV^ΔCME^ variant contains the complete NS1 gene, there is no need to supplement NS1 in downstream areas. This YFV^ΔCME^ variant can be used to map the whole monosynaptic projections (projectome) of a specific neuronal cell type [49].

Another effective monosynaptic anterograde tracer is the AAV1 vector, which was recently found to exhibit anterograde transneuronal spread at high titers [50]. AAV9 also exhibits similar properties, though at an even higher titer than AAV1. Other AAV serotypes, such as AAV5, AAV6, and AAV8, have not shown such properties. It is worth noting that the efficiency of AAV1-mediated transsynaptic tracing is relatively low compared to HSV. Therefore, this strategy requires the application of Cre or Flp recombinase to amplify the expression of downstream reporter genes, allowing a clearer identification and more efficient manipulation of postsynaptic neural pathways. Another caveat is that AAV1 can also retrogradely label presynaptic neurons (albeit at low efficiency) [50–52], which may confound the identification of postsynaptic neurons.

### Retrograde Transsynaptic Viral Tracers

Retrograde transsynaptic tracing is well-established and widely used in modern neuroscience research, benefiting from the development of the PRV and pseudotyped RV. It should be noted that PRV is not a rabies virus. Instead, similar to HSV, it belongs to the herpesviridae. It is an enveloped double-stranded DNA virus with a large payload for gene packages. The direction of PRV transport differs between distinct strains. The wild-type virulent strain, namely PRV-Becker, spreads bidirectionally (both anterogradely and retrogradely) between connected neurons, whereas another attenuated strain, PRV-Bartha, exhibits selective retrograde transport [53]. PRV-152, an engineered strain of PRV-Bartha by adding the *CMV-EGFP* cassette to the viral genome to allow GFP expression, is widely used for retrograde polysynaptic tracing [54, 55]. It should be cautioned that PRV is highly virulent and lethal to animals, usually leading to death in 3–4 days after intracerebral injection [56]. Even though PRV-Bartha is an attenuated strain, it only prolongs the life of the animals for several days [54]. Therefore, investigators should be careful and take security precautions when using PRV. Due to the nature of polysynaptic transport, PRV is an excellent tool for short-term retrograde tracing of multilevel neural circuits that terminate at the periphery.

Similar to HSV, however, it is difficult to differentiate the monosynaptic or polysynaptic connections of PRV-labeled neurons, owing to its uncontrollable transsynaptic spread. Another powerful vector, the engineered RV, came on stage to circumvent this limitation [57]. RV is an enveloped virus with a negative-sense single-stranded RNA genome that consists of only five genes [14]. Among the five genes, one encodes rabies glycoprotein (RG), the envelope protein that mediates the entry of RV into cells. Actually, wild-type RV spreads across multiple levels of synapses [58]; however, different from HSV, it naturally exhibits exclusively retrograde transport without inducing cytopathy or leakage to local glia [58, 59]. Retrograde monosynaptic tracing has been achieved by replacing the native G gene of RV with fluorescent reporter genes and pseudotyping this G-deleted RV with EnvA (EnvA-RVΔG), an avian virus envelope protein (Fig. 2). This pseudotyped EnvA-RVΔG loses the ability to infect mammalian cells and transfer to synaptically-connected neurons. When mammalian cells express exogenous tumor virus receptor A (TVA), a cognate avian receptor of EnvA, EnvA-RVΔG is able to selectively enter the neurons. However, it is unable to synaptically transfer unless the glycoprotein is exogenously complemented. Therefore, with helper AAVs expressing TVA and G in selected cell subpopulation in a Cre-dependent manner, EnvA-pseudotyped RVΔG is able to infect TVA-harboring cells, also known as starter cells. Moreover, with the complementation of G in these cells, RVΔG recovers the ability to transsynaptically spread to the upstream neurons that directly innervate the starter cells. Once the virus reaches the upstream (presynaptic) neurons, it fails to further spread to the second-order presynaptic neurons due to the absence of G, thus achieving monosynaptic retrograde tracing [60].

## Advances and Prospects in Viral Tracing

Recombinant neurotropic viruses have become the most potent and common tools in modern neuroscience research, regardless of neuronal morphology delineation and neural-circuit tracing. Since their initial discovery and application, each type of virus has been developed rapidly with much amazing progress during the past decades. In the following, we introduce some cutting-edge applications of the current recombinant viruses in neural-circuit tracing.

**Fig. 3:**
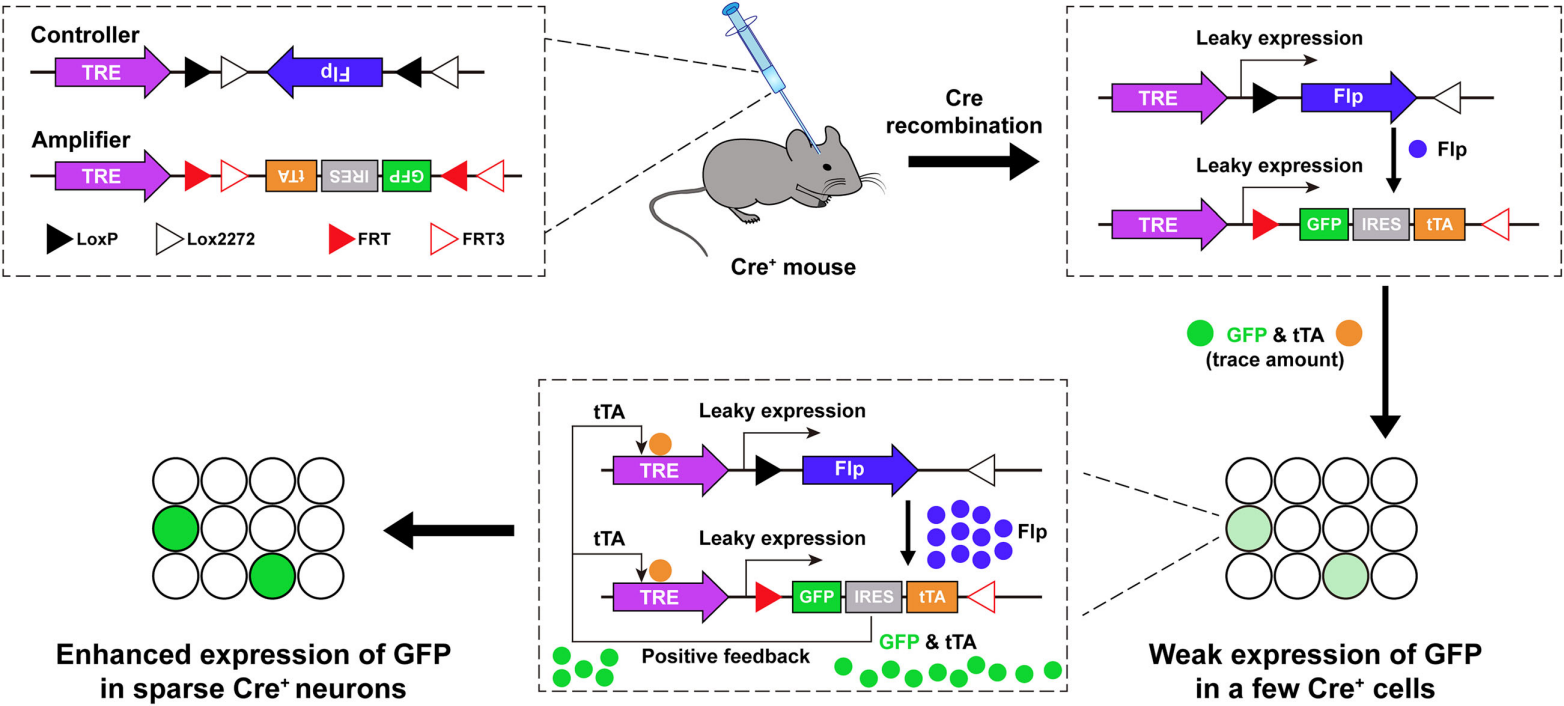
A dual-AAV system for sparse labeling of cell-type-specific neurons. Schematic of the design of the dual-AAV system for sparse labeling, comprising a “controller” and an “amplifier” AAV vector (upper left). When these two vectors are mixed and injected into the Cre-expressing mouse, leakage of TRE drives the weak expression of Flp in a few Cre^+^ neurons. Flp subsequently flips the GFP-IRES-tTA cassettes in the amplifier and drives the low level of GFP and tTA expression in these sparsely-labeled neurons (right panel). The small amount of tTA further binds to the TRE and potentiates the expression of Flp and GFP (lower middle), thereby triggering positive feedback to enhance GFP expression in sparse neurons (lower left). TRE, tetracycline response element; Flp, flippase; IRES, internal ribosome entry site; tTA, tetracycline trans-activator.

Among so many types of viruses, AAV is the most commonly used tool for transgene delivery into the host. It enables the labeling of local somas and distant axon terminals, which is indicative of the downstream projections of certain neuron populations. In addition, with the expression of the fluorescent reporter gene, AAVs delineate the neuronal morphology including soma, dendrites, and axon terminals. However, some neurons project distally to downstream regions to form long axonal tracts. The routinely used AAVs, due to the low intensity and non-uniformity of the fluorescence signals along long axons, are unable to provide a full view of the axon projection routes. Moreover, due to the high efficiency of AAV infection at the local injection site, it is difficult to differentiate individual neuronal morphology. A recently-developed dual-AAV system overcomes this limitation and achieves sparse and bright labeling of single neurons in a cell-type-specific manner [61]. This system includes a “controller” AAV vector which contains a tetracycline response element (TRE) promoter-driven, Cre-dependent, Flp expression cassette; and an “amplifier” vector, which contains a TRE promoter followed by an Flp-dependent *GFP-IRES-tTA* cassette. Since TRE is a bit leaky [62], it is able to drive low-level expression of downstream gene cassettes in the absence of the tetracycline trans-activator (tTA). When the “controller” and “amplifier” vectors are co-injected into the brain in a Cre-expressing mouse line, TRE-driven weak Flp expression (from the controller) only occurs stochastically in a few Cre-expressing neurons. It is in these neurons that Flp drives the recombination of the gene cassette in the amplifier, leading to minimal expression of the tTA protein due to the leakage of TRE. tTA subsequently binds to the TRE promoter and further potentiates the expression of Flp and tTA. This cascade reaction forms a positive feedback loop to enhance the GFP expression in only a few neurons, thereby resulting in sparse but bright labeling of single neurons (Fig. 3). The degree of labeling efficiency is tunable by adjusting the titer of the controller vector. Combined with fluorescent micro-optical sectioning tomography, a whole-brain reconstruction technology [63], this sparse labeling enables the complete exhibition of the morphology of cell-type-specific single neurons including their long axonal arborizations.

The retrograde tracer CAV-2 is usually used in combination with other vectors to achieve projection-specific tracing. For example, an AAV vector containing Cre-dependent gene cassettes, e.g., a fluorescent reporter gene, is injected into the candidate source region, while the CAV-2 carrying a Cre recombinase is injected into the putative downstream area. When the retrogradely transported CAV-2 reaches the neuronal soma, Cre recombinase drives the expression of the reporter gene, thereby selectively labeling neurons projecting to the downstream area [64]. However, the applicability of CAV-2 is limited by the restricted expression of CAR in the nervous system. A recent study overcame this limit using a receptor-complementation strategy by expressing CAR in the candidate projection neurons [65]. This leads to a substantially increased retrograde-labeling efficiency of CAV-2. Moreover, by designing Cre-dependent CAR deletion (Cre-OFF) along with simultaneous expression of fluorescent reporters or channelrhodopsin (Cre-ON), this strategy not only removes CAR from retrogradely-labeled neurons to avoid potential interference with normal neuron function, but also provides a way to structurally and functionally characterize the neural circuits.

**Fig. 4:**
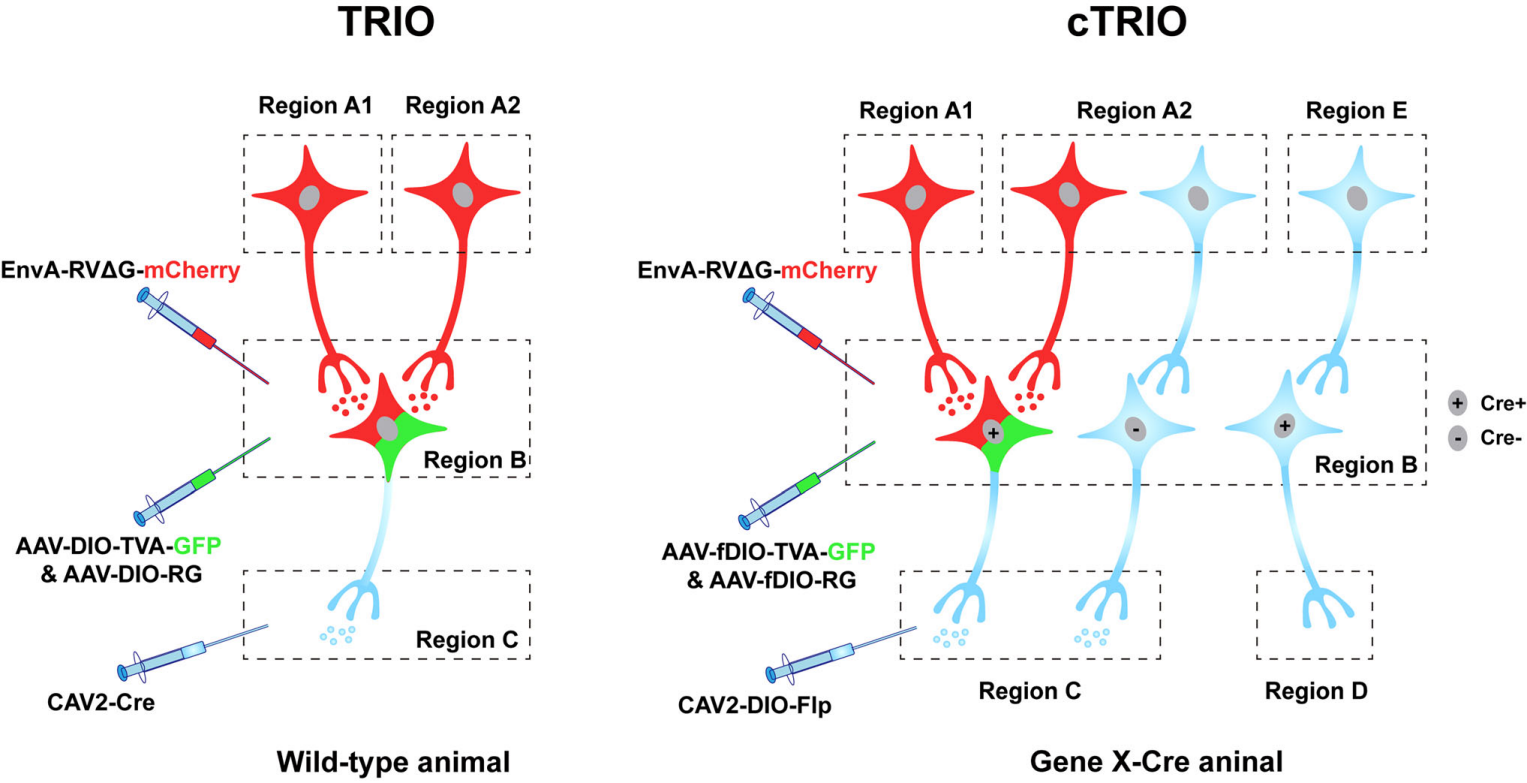
Viral strategies of TRIO and cTRIO. TRIO combines the application of CAV-2 and RV, thus allowing projection-specific retrograde tracing within three-node neural circuits (from regions A to C *via* B). cTRIO further combines genetic approaches to allow mapping cell type- and projection-specific neural circuits (from regions A to C *via* Cre^+^ cells in B). TRIO, tracing the relationship between input and output; cTRIO, cell-type-specific TRIO; DIO, double-floxed inverse open reading frame; fDIO, Flp-controlled DIO.

The EnvA-coated RVΔG system is widely used, not only to trace the monosynaptic inputs to specific neuronal populations [66, 67], but also to manipulate the neural circuits in combination with other viral tools expressing elements such as channelrhodopsin and designer receptors exclusively activated by designer drugs (DREADDs) [68]. Recently, ingenious tracing strategies combining the application of CAV-2 and RVΔG, called TRIO (tracing the relationship between input and output) and cTRIO (cell-type-specific TRIO), which are able to achieve three-node (e.g. A–B–C) circuit tracing, have been developed [69] (Fig. 4). In TRIO, AAV helpers carrying Cre-dependent TVA receptors and RG are delivered into region B, while CAV-2 expressing Cre recombinase is injected into the downstream region C. CAV2-Cre virus retrogradely spreads to region B and drives the expression of TVA and RG only in the neurons projecting to region C. EnvA-pseudotyped RVΔG is then allowed to infect these projection-specific neurons and is transsynaptically transported to the presynaptic neurons in upstream region A. In cTRIO, this three-node tracing approach has been applied in transgenic mice and enables the identification of the inputs and outputs of specific cell types in region B. Specifically, AAV helpers injected into region B are replaced by Flp-dependent TVA and RG, while CAV-2 expressing Flp recombinase in a Cre-dependent manner is delivered into the downstream region C. In this way, in region B, only neurons that contain Cre and innervate region C can express TVA and RG, allowing the subsequent RVΔG-mediated transsynaptic labeling of neurons in region A.

Although this intersectional strategy is now commonly used for circuit mapping between multilevel neurons, it still has some limitations [70]. First, the widely used RV strain SAD-B19 is an attenuated strain with relatively low efficiency of synaptic transfer [71]. Second, despite reduced cytotoxicity of the attenuated strain, RV infection leads to cell death within 1–2 weeks, thereby hindering its use in the long-term functional manipulation of neural networks. Fortunately, recent studies have generated a new G-deleted RV strain (CVS-N2CΔG) with enhanced transsynaptic transfer and reduced cytotoxicity, which partly overcome these two limitations [72, 73]. Another study has also developed a self-inactivating RVΔG (SiR), which switches off in primary infected cells *via* proteasomal degradation that disrupts the viral transcription-replication cycle. Since SiR also carries a Cre element, it allows permanent genetic access to the traced cells but prevents neuronal toxicity [74].

**Fig. 5:**
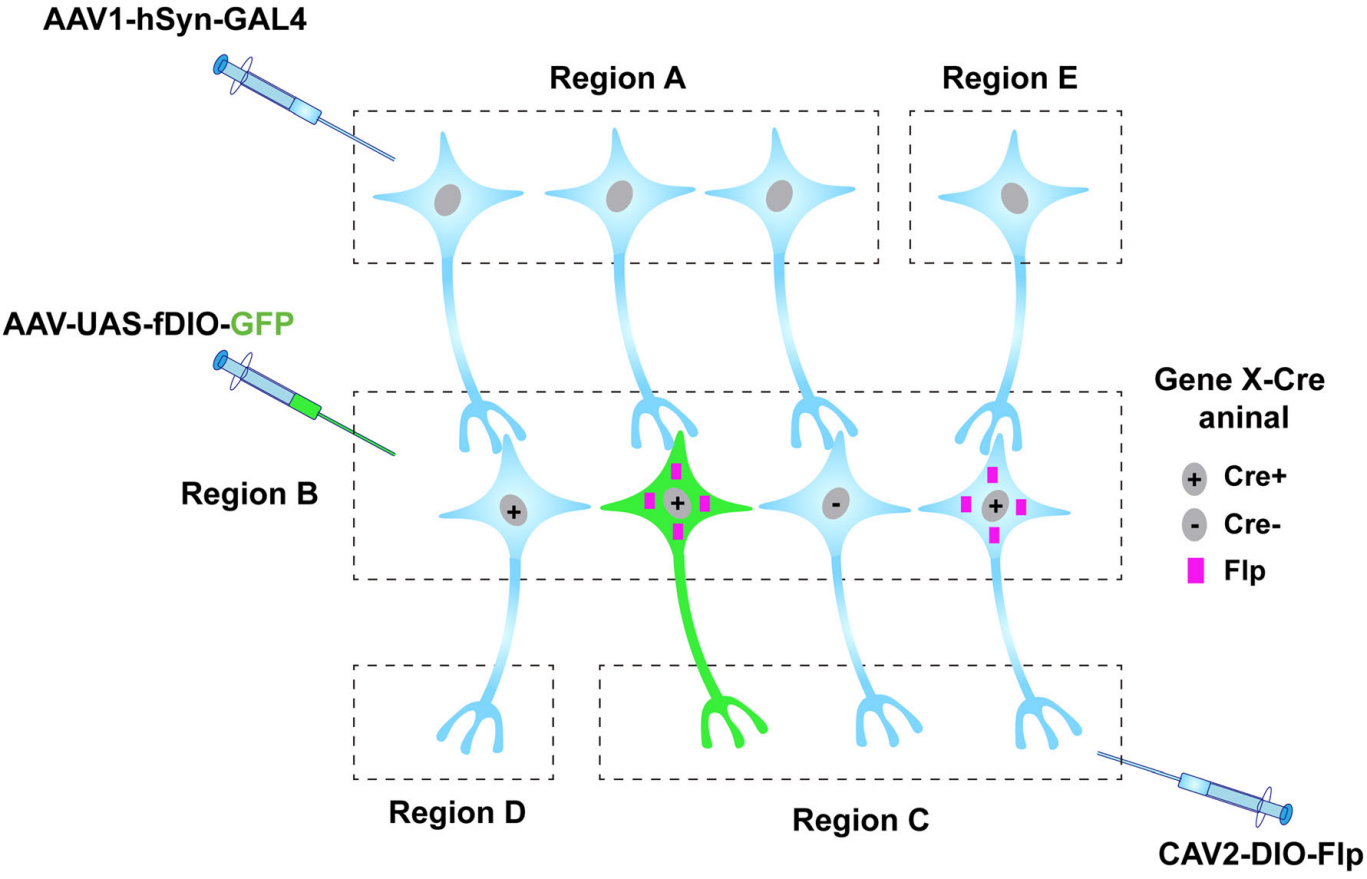
Intersectional strategies to define and access more specific cell types. With the combination of multiplex recombinase systems, including GAL4-UAS, Cre-LoxP, and Flp-FRT, a more specific subset of neurons can be defined by their input, output, and molecular identity. The schematic shows that neurons expressing Cre and projecting to region C contain Flp. However, only neurons that receive input from region A (shown in green) can express GAL4, which binds to the UAS promoter and initiates the downstream expression of the virus injected into region B. In this case, a specific cell type expressing Cre, which is innervated by certain inputs and projects to putative downstream targets, can be accessed.

While current viral tools still have limitations in different aspects, the combinatorial use of viruses with diverse genetic strategies would expand their application. As described above, engineered viruses can label the neural circuits of interest in a cell type-specific and projection-specific manner, in combination with different recombinase systems. However, it is insufficient to define and get access to a function-specific neuronal subset based on one marker gene, as neurons are diverse with hundreds of subcategories. Intersectional strategies using multiplex recombinase systems allow viruses to get genetic and functional access to a more specific cell subtype [75]. In a recent study, a Cre- and Flp-dependent virus was delivered to a double transgenic mouse, in which Tac1-positive neurons expressed Cre and GABAergic neurons expressed Flp, thereby specifically labeling and manipulating the Vgat and Tac1 double-positive neurons [76]. Another study used viruses expressing recombinases driven by cell subclass-specific enhancers in new three-color reporter mice, Ai213, allowing specific and simultaneous labeling of three distinct cell subsets in the mouse cortex [77]. In the future, by virtue of versatile recombinases and viral systems, we may be able to label even more specific subsets of neurons (Region B in Fig. 5) based on input- and projection-tagging strategies (Fig. 5). For example, there are two different cell populations (Cre^+^ and Cre^−^) in region B. By injecting CAV2-DIO-Flp in B’s putative downstream area (Region C), Cre^+^ cells projecting to C express Flp recombinase. However, this particular population of cells is usually innervated by multiple upstream inputs. After injecting the anterograde transsynaptic AAV1 vector carrying GAL4 into a presynaptic region (A), neurons (in B) innervated by region A express GAL4. By further injecting the UAS (upstream activating sequence)-driven AAV vector expressing Flp-dependent GFP into the local region (B), we are able to specifically label Cre^+^ cells (shown in green) innervated by region A but projecting to C. By this means, a more specific subset of neurons, characterized by their specific input, output, and molecular identity, can be genetically accessed (Fig. 5).

Currently, viral tracers are mostly delivered through an invasive craniotomy or injection into peripheral tissue. For intersectional labeling strategies, several different types of viruses are injected into different target regions, which unavoidably leads to damage or trauma. Systemic delivery, mostly *via* intravenous injection, provides a simpler and non-invasive alternative for virus delivery to the central and peripheral nervous systems [78, 79]. The newly-developed AAV variant AAV.CAP-B10 exhibits high specificity and efficiency to target neurons in the brain after intravenous delivery, with rare accumulation in the liver [80]. Although intravenous injection is more commonly used for gene therapy, it is plausible to suppose that systemic delivery could also be used in viral tracing to access specific cell types. For example, by locally applying focused ultrasound to open the blood-brain barrier, systemically-delivered viruses are able to enter this target region [81]. Moreover, adding cell-type-specific promoters into the viral genome, using the recombinase systems, or engineering the viral capsids according to the cell and tissue tropism, also helps to achieve the cell-type-specific entry of systemically-delivered viruses.

## Conclusions

Engineered viruses have become the most powerful tools in the technical arsenal of modern neuroscience. The ideal viral tools would be avirulent with a larger capacity for transgene packaging, easier and non-invasive delivery, and greater specificity to target cells but wider applicability across species [82]. In addition to the traditional modification of the viral genome and capsid/envelope, new methods, including M-CREATE (multiplexed Cre recombination-based AAV targeted evolution) [83] and machine learning-guided design [84], have been developed to screen and engineer new recombinant viruses with wider applicability. As the unknown mechanisms of the virus are gradually unveiled, we believe that more suitable viral tools will be developed in the near future and accelerate the advances of neuroscience.
